# Small-size temperature/high-pressure integrated sensor via flip-chip method

**DOI:** 10.1038/s41378-024-00723-3

**Published:** 2024-07-23

**Authors:** Mimi Huang, Xiaoyu Wu, Libo Zhao, Xiangguang Han, Yong Xia, Yi Gao, Zeyu Cui, Cheng Zhang, Xiaokai Yang, Zhixia Qiao, Zhikang Li, Feng Han, Ping Yang, Zhuangde Jiang

**Affiliations:** 1 State Key Laboratory for Manufacturing Systems Engineering, International Joint Laboratory for Micro/Nano Manufacturing and Measurement Technologies, Xi’an Jiaotong University, Xi’an, 710049 China; 2 Xi’an Jiaotong University (Yantai) Research Institute for Intelligent Sensing Technology and System, Xi’an Jiaotong University, Xi’an, 710049 China; 3 Shandong Laboratory of Yantai Advanced Materials and Green Manufacturing, Yantai, 265503 China; 4 School of Mechanical Engineering, Xi’an Jiaotong University, 710049 Xi’an, China; 5 School of Instrument Science and Technology, Xi’an Jiaotong University, 710049 Xi’an, China; 6 Chongqing Key Laboratory of Micro-Nano Systems and Intelligent Sensing, Chongqing Academician Workstation, Chongqing 2011 Collaborative Innovation Center of Micro/Nano Sensing and Intelligent Ecological Internet of Things, Chongqing Technology and Business University, Nan’an District, Chongqing, 400067 China; 7 Xi’an Aerospace Yuanzheng Fluid Control Co. Ltd., Xi’an, 710049 China

**Keywords:** Sensors, Engineering

## Abstract

Hydraulic technology with smaller sizes and higher reliability trends, including fault prediction and intelligent control, requires high-performance temperature and pressure-integrated sensors. Current designs rely on planar wafer- or chip-level integration, which is limited by pressure range, chip size, and low reliability. We propose a small-size temperature/high-pressure integrated sensor via the flip-chip technique. The pressure and temperature units are arranged vertically, and the sensing signals of the two units are integrated into one plane through silicon vias and gold–gold bonding, reducing the lateral size and improving the efficiency of signal transmission. The flip-chip technique ensures a reliable electrical connection. A square diaphragm with rounded corners is designed and optimised with simulation to sense high pressure based on the piezoresistive effect. The temperature sensing unit with a thin-film platinum resistor measures temperature and provides back-end high-precision compensation, which will improve the precision of the pressure unit. The integrated chip is fabricated by MEMS technology and packaged to fabricate the extremely small integrated sensor. The integrated sensor is characterised, and the pressure sensor exhibits a sensitivity and sensitivity drift of 7.97 mV/MPa and −0.19% FS in the range of 0–20 MPa and −40 to 120 °C. The linearity, hysteresis, repeatability, accuracy, basic error, and zero-time drift are 0.16% FS, 0.04% FS, 0.06% FS, 0.18% FS, ±0.23% FS and 0.04% FS, respectively. The measurement error of the temperature sensor and temperature coefficient of resistance is less than ±1 °C and 3142.997 ppm/°C, respectively. The integrated sensor has broad applicability in fault diagnosis and safety monitoring of high-end equipment such as automobile detection, industrial equipment, and oil drilling platforms.

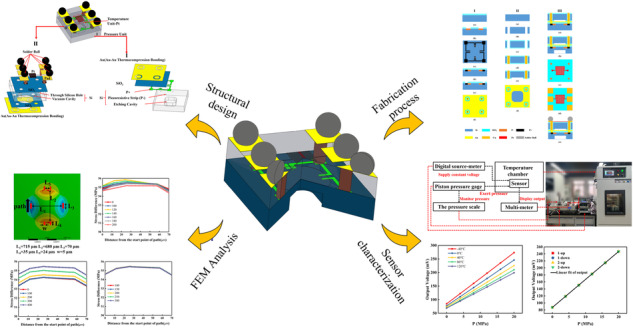

## Introduction

Hydraulic fluid transmission is one of the most widely utilised transmission technologies in aerospace, engineering, robotics, and other industries. Hydraulic systems have evolved to be lighter in weight, smaller in size, with a higher pressure range, higher power density, and more intelligent, with the need for higher reliability and safety. The detection of hydraulic system faults is critical for avoiding system failure. As a typical hydraulic system, an electro-hydrostatic actuator (EHA) is an important growth path with advantages such as small size, high hydraulic output force, and flexible control ^1–4^. However, owing to the lack of multi-parameter in-situ testing and the bulk and quality requirements of the testing parts, the ability to predict EHA faults is limited.

The high-level integration of the EHA places additional demands on system safety. In a hydraulic system, the load size determines the operating pressure. It must operate within a specified pressure range to ensure the system operates safely and normally. Oil is utilised as the hydraulic transmission medium to achieve energy conversion. High oil temperatures cause a decrease in the fluid viscosity, leakage, accelerated ageing of parts, and other difficulties that result in various hydraulic system failures^5^. Hence, temperature and pressure are two critical parameters related to hydraulic system safety with the miniaturisation and intelligent development of hydraulic systems. It is necessary to detect the temperature and pressure simultaneously to monitor system safety and avoid the occurrence of faults. Therefore, temperature and pressure-integrated sensors have broad application prospects and have garnered significant attention. Integrated sensors can be divided into flexible, fibre-optic, and silicon-based sensors based on the sensing materials. Flexible sensors are mostly used in the fields of robot skin and biomedicine, and their pressure–temperature range is relatively small^6–10^. Although optical fibre sensors have the advantages of anti-interference, electrical insulation, corrosion resistance, and long-distance signal transmission, their signal processing requires special regulators, which increases the cost of post-processing^11–15^. However, sensors made of flexible and optical materials have problems such as a non-standard fabrication process and poor repeatability.

Based on MEMS techniques, silicon-based temperature/pressure integrated sensors have the features of small size, high repeatability, and a large linear measuring range and are widely utilised in industrial applications. Integrating sensors can be divided into two categories based on integration methods. The first is chip-level stacking^16^. Although flexible in integration, it greatly increases the cost and packaging size owing to its low efficiency and difficulty in small-die handling. The second is monolithic integration. According to the application requirements, multisensors are fabricated on the same silicon substrate using MEMS techniques.

The pressure units in the integrated sensors designed by Lin^17^ and Chien^18^ were based on the capacitive effect, and the sensitivities in the ranges of 20–120 kPa and 20–100 kPa were 0.969 fF/kPa and 0.200 fF/kPa, respectively. Lin’s resistance temperature detector had a sensitivity of 0.26%/°C in the range of 25–85 °C. Chien used a diode temperature sensor, and the sensitivity was 0.82 mV/°C at 25–85 °C. Although capacitive pressure sensors have high temperatures and long-term stabilities, they are sensitive to the parasitic capacitance introduced by packaging structures, measurement mediums, and circuits.

Cheng^19^ designed an integrated sensor with a resonant pressure unit and a Pt resistor temperature unit. Two parts with differential and static pressures were designed for the pressure sensing units. The sensitivity of the differential pressure part was 79.76 Hz/kPa in the range of 0–100 kPa, and the measurement accuracy of the static pressure component was 0.02% FS in the range of 110–200 kPa. The temperature unit was a Pt resistor with a sensitivity of 6.22 Ω/°C in the range of −20 to 60 °C. Although the resonant type is highly precise, the fabrication process is complicated, and the pressure range is narrow.

The integrated sensor designed by Li^20^ used polysilicon for both pressure and temperature sensors. The pressure range was 0–450 kPa, and the full output was 45.9 mV, the linearity was ±0.84% FS. The temperature coefficient of resistance (TCR) of the temperature sensor was −578 ppm/°C. The final chip size was 2.5 × 2.5 × 0.84 mm^3^.

Zhao^21^ designed an integrated sensor in which both pressure and temperature used the piezoresistors. The sensitivity of the pressure sensor was 0.020 mV/V/kPa with a non-linearity of 0.4% FS in the range of 0–200 kPa. The sensitivity of the temperature sensor was 5.617 Ω/°C with a non-linearity of 0.48% FS in the range of −30 to 150 °C. The final chip size was 4 × 6 × 0.9 mm^3^.

The existing monolithic integrated sensors are tiled, and all the sensing units are arranged in a plane. Consequently, the fabrication process should be compatible with every sensing unit, however, the performance of the sensing units may inevitably be lost. In addition, because the chips are packaged by wire-bonding, the size of the packaged sensor is large, and the reliability of the electronic connection is poor in harsh environments, such as strong vibrations.

Here, we propose a vertically integrated temperature/pressure sensor based on leadless packaging techniques. The integrated sensor uses piezoresistors and platinum thin-film resistors as the pressure and temperature-sensing elements, respectively. The pressure and temperature units are arranged vertically and closely bonded with through-silicon vias (TSV) and Au–Au thermocompression bonding processes, ensuring electrical connections and saving planar space. A square diaphragm with rounded corners for pressure sensing is designed and optimised based to the numerical simulation results of the structure’s diaphragm stress and natural frequency. Finally, an integrated sensor is fabricated, and its performance is characterised. The results reveal that a sensor with a small size has high sensitivity, linearity, repeatability, and basic error.

## Material and methods

### Model and structure

**Fig. 1:**
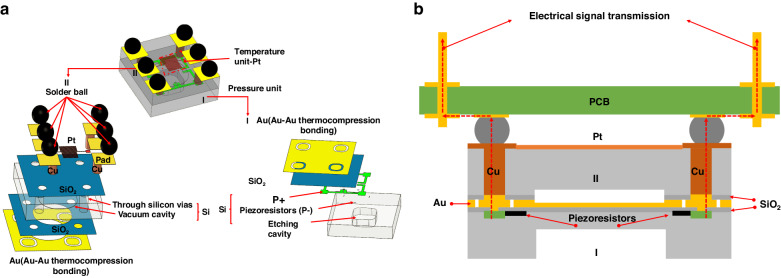
Structure and electrical signal transmission. **a** Structure of pressure/temperature integrated sensor-based flip chip method. **b** Vertical electrical signal transmission path

The designed integrated sensor is shown in Fig. 1a. There are two components: a pressure-sensing unit and a temperature-sensing unit. To reduce the lateral size of the chip and avoid mutual interference, two units are placed on Silicon Wafers I and II. Three types of elastic diaphragms are used in the pressure sensors: circular, rectangular, and square. A circular diaphragm is not conducive to mass production. The maximum stress area of the rectangular diaphragm is located at the centre of the elastic diaphragm, and the maximum stress area of the square diaphragm is at the centre of the four sides of the elastic diaphragm. In the case of the same short side, the stress difference of the rectangular diaphragm is smaller than that of the square diaphragm; Therefore, the square diaphragm is finally selected to obtain a higher sensitivity. A rounded-corner design is added to the square diaphragm film to further improve the sensitivity and natural frequency of the pressure sensor.

The piezo resistors are arranged in an appropriate position to maximise the pressure-induced resistive change and are connected by heavy doping to form a Wheatstone full bridge. Compared with conventional designs, the fabrication process can be simplified by omitting the addition of metal and insulating layers. In addition, the internal stresses around the piezoresistors are reduced because of the fewer covering layers.

A meander-shaped platinum resistor is designed on Silicon Wafer II to detect the ambient temperature. To integrate the electrical signals of the two units into one plane, the electrical signals in the pressure sensor are transferred to the same side of the platinum resistor through electroplated copper columns. Simultaneously, a vacuum cavity is etched on Silicon Wafer II such that the pressure-sensitive diaphragm can move freely.

Silicon Wafers I and II are integrated into a reliable whole via Au–Au thermocompression bonding. The same thermal expansion coefficients for Silicon Wafers I and II are utilised to reduce the generation of internal stress during bonding. Six pads are designed for ball planting instead of conventional wire bonding to realise electronic connections. The transmission path of the electrical signal is shown in Fig. 1b. Compared with the traditional wire-bonding structure, the transmission distance is greatly reduced, and the vertical transmission effectively improves the transmission efficiency of the electrical signal.

### FEM analysis

**Fig. 2:**
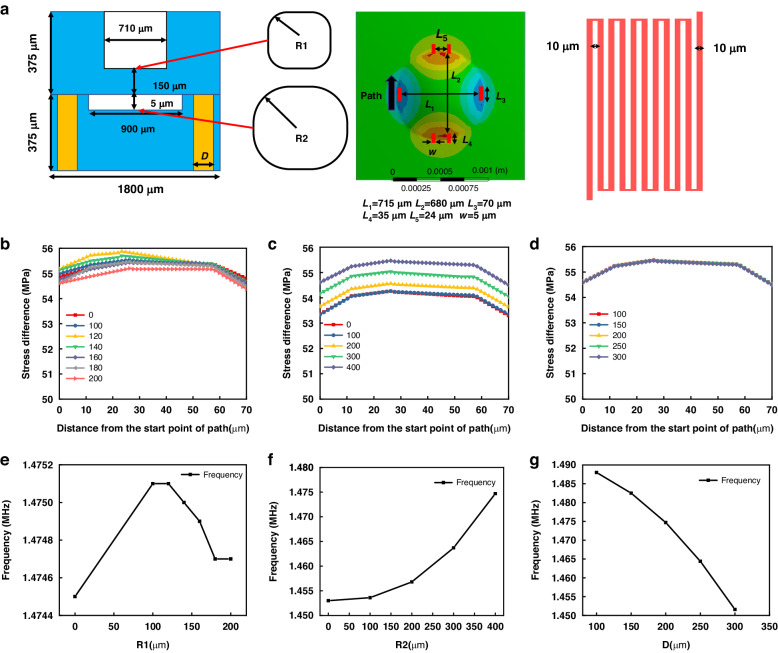
Stress distribution and natural frequency. **a** Dimensional schematics of the pressure sensor and temperature sensor. **b** Effects of *R1* on stress difference distribution. **c** Effects of *R2* on stress difference distribution. **d** Effects of *D* on stress difference distribution. **e** Effects of *R1* on natural frequency. **f** Effects of *R2* on natural frequency. **g** Effects of *D* on the natural frequency

The critical dimensions of the integrated sensor were optimised with the ANSYS Workbench. The material properties of Si were set as density *ρ* = 2330 kg/m^3^, elastic modulus *E* = 1.69E11 Pa, and Poisson’s ratio *ν* = 0.28^22^. Fixed constraints were applied to the four peripherals of the square-sensitive diaphragm, and a pressure of 20 MPa was applied to Wafer I. Then, stress and modal analyses of the pressure unit in the integrated sensor were conducted. The identified sizes of the sensor chips in Fig. 2a are the values obtained from simulation and fabrication. Because the structure is completely symmetrical, the design effect can be reflected by analysing the stress difference in the path. The locations and dimensions of the piezoresistors are illustrated the second figure in Fig. 2a.

To further improve the sensor’s performance, the rounded corners radii of the etching cavity *R*1, vacuum cavity *R*2, and diameter *D* of the TSV were analysed and optimised. The setting of *R*1 was designed to ensure that the reaction gas in the sharp corner area could not exchange well during the dry-etching process, which affected the etching pattern and efficiency. As illustrated in Fig. 2b, the stress distributions on the piezoresistor were calculated under different rounded corner radii with the range of 0–200 μm. The results revealed that the stress increased in the range of 0–120 μm and slightly decreased in the range of 140–200 μm. In addition, the stress distributions on the piezoresistor were not uniform in the *R*1 range of 0–160 μm. The influence of *R1* on the natural frequency is not monotonic but has a peak value. As illustrated in Fig. 2e, when *R1* was 100 and 120 μm, the natural frequency reached the maximum, then the natural frequency decreased continuously, and the frequency tended to be stable until 180 μm. However, the addition of *R1* increases the natural frequency to varying degrees. Considering the uniformity of stress distribution, *R1* was finally selected as 180 μm. The design of *R*2 primarily considers the bonding area under the condition of a small overall chip size. The larger the bonding surface, the greater the chance of increasing the bonding strength. A rounded corner was added to the squares to increase the bonding area. Therefore, a rounded corner design was added without losing performance to increase the bonding area and improve the bonding quality. The stress distributions corresponding to different rounded corners are presented in Fig. 2c. The results revealed that an increased rounded corner size led to a monotonic increase in stress, and the uniformity of the distribution remained. The influence of *R2* on the natural frequency was monotonous, and the natural frequency increased with increasing *R2* as shown in Fig. 2f. The final size of *R*2 was 400 μm.

The final important parameter *D* is the diameter of TSV. As illustrated in Fig. 2d, the stress distributions of the piezoresistor along the path were obtained with different corresponding diameters *D*. The simulation results confirm that the value of diameter *D* had little effect on the stress distribution. Simultaneously, the natural frequencies of sensor chips with different diameters *D* were simulated, as illustrated in Fig. 2g. As the value of diameter *D* increased, the natural frequencies decreased. The final diameter *D* was set to 200 μm according to the current fabrication process level.

There was a detailed description of the temperature sensor design in a previous article^23^. We have not replicated this here. The interval and finish line widths were both fixed at 10 μm as illustrated in the third figure in Fig. 2a. Because the piezoresistive pressure sensor is sensitive to temperature, a temperature sensor at the same position can measure the temperature in real-time to guide the temperature compensation of the pressure sensor, thereby improving the precision of the pressure sensor in the full temperature range. The compensation method was described in detail in our recently published article^24^.

### Working principle

**Fig. 3:**
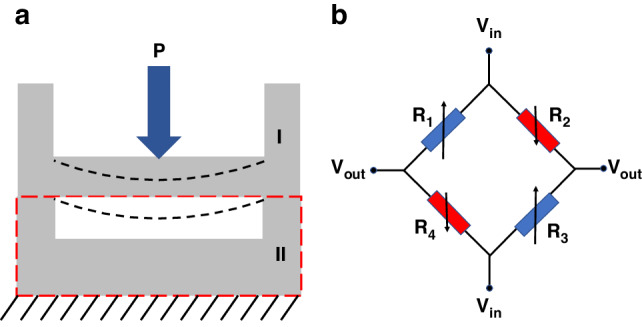
Working principle of pressure sensor. **a** Deformation of back-loaded. **b** Wheatstone full-bridge

The pressure unit of the integrated sensor chip was backloaded, as illustrated in Fig. 3a. This design lowered the requirements for the pressure medium compared to the front-loaded. Design four piezoresistors were placed at the positions with the maximum stress difference to form the Wheatstone full bridge, as illustrated in Fig. 3b. When pressure is applied to the pressure diaphragm, the resistance values of the four piezoresistors change, as illustrated in Eq. 1.

1 $$\begin{array}{c}\frac{\varDelta {R}_{1}}{{R}_{1}}=\frac{\varDelta {R}_{3}}{{R}_{3}}=\frac{1}{2}{\pi }_{44}({\sigma }_{l1}-{\sigma }_{t1})\\ \frac{\varDelta {R}_{2}}{{R}_{2}}=\frac{\varDelta {R}_{4}}{{R}_{4}}=\frac{1}{2}{\pi }_{44}({\sigma }_{l2}-{\sigma }_{t2})\end{array}$$

where *π*_44_ is the piezoresistive coefficient P-type silicon. The longitudinal stress on *R*_1_ and *R*_3_ is *σ*_l1_, the transverse stress is *σ*_t1_, the longitudinal stress on *R*_2_ and *R*_4_ is *σ*_l2_, and the transverse stress is *σ*_t2_.

Owing to the change in the output voltage, the relationship between the measured pressure and the output voltage is linear. The change law is expressed by Eq. 2^25^.

2 $${V}_{{out}}=\frac{({R}_{2}{R}_{4}-{R}_{1}{R}_{3})}{({R}_{1}+{R}_{2})({R}_{3}+{R}_{4})}{V}_{{in}}=\frac{\varDelta R}{R}{V}_{{in}}=\frac{{\pi }_{44}}{2}{\sigma }_{lt}{V}_{{in}}$$

where *σ*_*lt*_ is the stress difference between longitudinal stress and transverse stress. *V*_in_ is the input voltage.

The sensitivity is expressed by Eq. 3.

3 $$S=\frac{{V}_{{out}}({P}_{\max })-{V}_{{out}}({P}_{\min })}{{P}_{\max }-{P}_{\min }}$$

where *P*_max_, *P*_min_ are the maximum and minimum pressure, *V*_*out*_(*P*_max_) and *V*_*out*_(*P*_min_) are the output under the maximum and minimum pressure. According to the simulation results and Eqs. 2 and 3, the theoretical full-scale output and sensitivity are 154.70 mV and 7.74 mV/MPa (with *π*_44_ = 112.5e−11 Pa^−1^, *V*_*in*_ = 5 V, *σ*_*lt*_ = 55 MPa.).

Platinum was selected as the sensing material for the temperature sensor because of its stable chemical properties, excellent linearity, and precision^26–29^. The operation of the temperature sensor unit is based on the theory of metal thermal resistance. Eq. 4 illustrates the temperature properties of a platinum resistor and how they change as a function of temperature.

4 $$\left\{\begin{array}{l}{R}_{t}={R}_{0}[1+At+B{t}^{2}+C{t}^{3}(t-100)]\,\,\,\,\,\,\,\,(-200\le t\le 0)\\ {R}_{t}={R}_{0}(1+At+B{t}^{2})\,\,\,\,\,\,\,\,\,\,\,\,\,\,\,\,\,\,\,\,\,\,\,\,\,\,\,\,\,\,\,\,\,\,\,\,\,\,\,\,\,\,\,\,\,\,(0\le t\le 630)\end{array}\right.$$

where *R*_0_ is the resistance of platinum at 0 °C, and *R*_*t*_ is the resistance of platinum at different temperatures. *A*, *B*, and *C* are the first-, second-, and third-order temperature coefficients, respectively. Because the values of *B* and *C* are negligible, the change in the resistance of the platinum resistor is proportional to the temperature.

## Results and discussion

### Fabrication and packaging

**Fig. 4:**
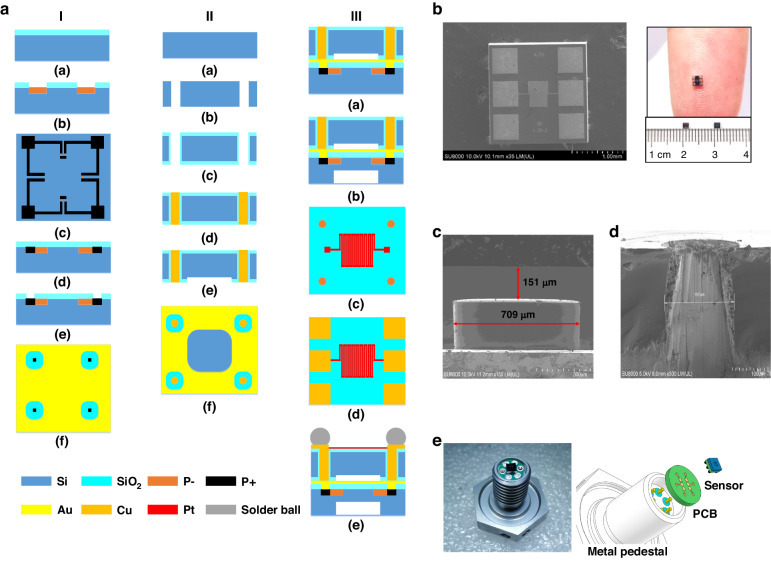
Fabrication processes and integrated chip and Package. **a** Fabrication process. **b** Chip image. **c** SEM images of pressure diaphragm size. **d** SEM images of TSV cross section. **e** Packaged sensor

The fabrication process is demonstrated in Fig. 4a. The Au–Au thermocompression bonding of two silicon wafers fabricated the integrated sensor. The two silicon wafers are N-type (100) crystal planes with resistivity of 1–10 Ω cm and thickness of 375 μm. Because bonding requires the flatness of the silicon wafer, the two silicon wafers must be ultra-flat. The silicon wafers of the pressure unit, TSV process, and bonded silicon wafer were Wafers I, II, and III, respectively.

The fabrication process of Silicon Wafer I was as follows.

(a) The silicon wafer was cleaned, and a layer of SiO_2_ was fabricated on the front of the silicon wafer by plasma-enhanced chemical vapour deposition as a mask layer for ion implantation.

(b) A piezoresistor pattern was formed by photolithography, and four piezoresistors were formed by boron ion implantation. The implantation dose was 5e14 cm^−^^2^. The piezores-istors were oriented in 〈110〉 direction.

(c) The SiO_2_ layer was removed and another SiO_2_ layer was regrown for heavy doping masking. Heavy doping areas were formed via photolithography. These heavily doped areas had two functions: to connect with the piezoresistors to form a Wheatstone full bridge and to form an ohmic contact with the metal. The implantation dose was 5e14 cm^−2^.

(d) All the SiO_2_ layers were removed, and the silicon wafer was annealed to repair the damage caused by ion implantation. The annealing condition was 1000 °C for 60 min under N_2_ atmosphere. A layer of 300 nm SiO_2_ was grown by low pressure chemical vapour deposition for insulation.

(e) Lead holes were etched to combine the metal and heavily doped areas.

(f) The sputtered metal played two roles. One was to form a good ohmic contact with the heavily doped areas to extract the chip signal, and the other was utilised as the Au–Au thermocompression bonding medium. Therefore, Ti/Au metal was sputtered on the front, and lithography and etching techniques were performed to ensure an Au–Au thermocompression bonding area.

Silicon Wafer II was utilised in the TSV process, and the process was as follows.

(a) The silicon wafer was cleaned with a certain proportion of H_2_SO_4_ and H_2_O_2_, then rinsed with deionized water, and finally blown dry with nitrogen.

(b) Photolithography and deep silicon etching of circular holes with a diameter of 200 μm and depth of 375 μm for the preparation of TSV.

(c) SiO_2_ layers were fabricated on both sides of the silicon wafer and inside the circular holes utilising a thermal oxygen process to ensure device insulation, and the thickness of SiO_2_ was 2 μm.

(d) Circular holes were filled with copper via electroplating to achieve TSV.

(e) A vacuum cavity was etched by ICP (Inductively Coupled Plasm) etching to ensure that the pressure-sensitive diaphragm had moving space, and the depth was 5 μm.

The bonded Silicon Wafer III process was as follows.

(a) Silicon Wafers I and II were combined by Au–Au thermocompression bonding technology such that the signal could be directed to the top surface by electroplating copper.

(b) The pressure measuring unit was etched in the back cavity to form a 150 μm pressure-sensitive diaphragm. Since ICP etching was used, the design of the rounded corner was beneficial to the gas reaction and the elimination of the product, thus ensuring the patterning and improving the uniformity of the etching.

(c) Ti/Pt layers were obtained by a stripping process to form a platinum resistor as a temperature sensor for sensing temperature change.

(d) Welding pads were prepared via electroplating. Four corner pads were utilised for the pressure sensor, and the middle pads were utilised for the temperature sensor. All electrical signals were concentrated in one plane.

(e) Solder balls were placed on welding pads to facilitate the formation of stable electrical connections.

The fabricated integrated sensor chip is presented in Fig. 4b with the size of 0.8 × 0.8 × 0.75 mm^3^. The SEM images of the pressure diaphragm size and TSV cross section are displayed in Fig. 4c, d, and the machined dimensions match the design dimensions. To realise a leadless package, a package scheme was designed, as shown in Fig. 4e. The sensor chip was firmly connected to six welding pads on the PCB with solder balls, and the six holes of the PCB were assembled with the corresponding pins on the metal base to complete the package. The dimensions of the sensor head are M8 × 10 mm. Compared with the conventional package utilising gold wire ball welding, solder balls and PCB connections effectively avoided conventional circuit-breaking faults caused by harsh environments, such as strong vibrations. Therefore, the provided package was reliable, and the size of the integrated sensor was small.

### Experimental test results

**Fig. 5:**
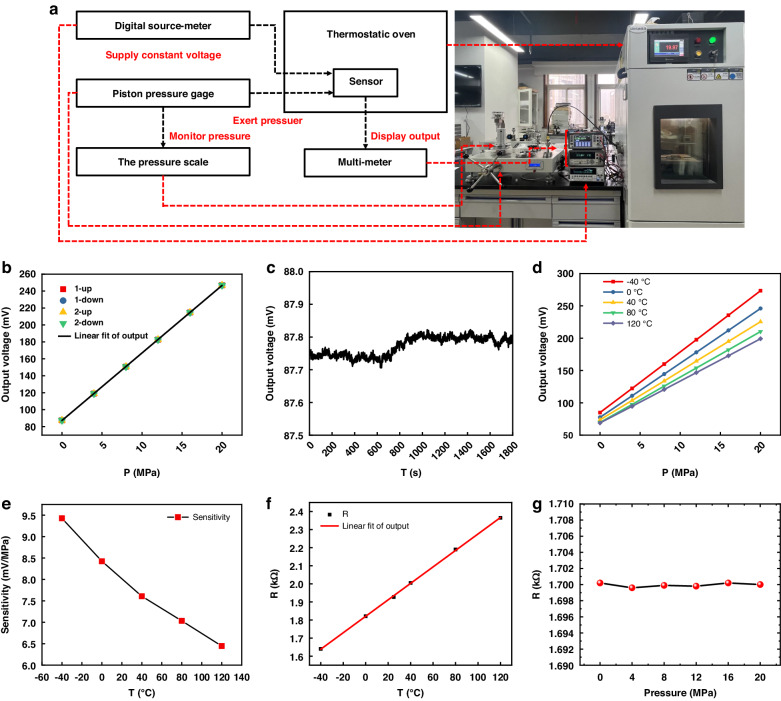
Experimental platform, the performance of the sensor. **a** Centrifugal test system for static performance testing. **b** Outputs of pressure unit at 25 °C. **c** Zero offsets of pressure unit. **d** Outputs of pressure unit at different temperatures. **e** Sensitivity of pressure unit at different temperatures. **f** Outputs of temperature unit. **g** Temperature sensor output under different pressures

An experimental platform was built to test the performance of the integrated sensor, as illustrated in Fig. 5a. The sensor was placed in a temperature chamber (FDW 701 P) with a control accuracy of ±0.5 °C to maintain the different temperatures. The piston pressure gauge (CW-600T) with control accuracy of 0.02% provided different pressures in the range of 0–20 MPa utilising weights with a pressure interval of 4 MPa. During the tests, a source table (Keithley 2612B) provided a constant voltage of 5 V to the pressure unit of the integrated sensor. A multimeter (Keithley DAQ6510) detected the changes in the output voltage of the pressure sensor, whereas another multimeter recorded the changes in the resistance of the temperature sensor.

The performance of the pressure unit in the integrated sensor was tested at 25 °C with the built experimental platform. Two rounds of travel tests were conducted on the sensor at 0–20 MPa. The experimental data are presented in Table 1, and the test curves are shown in Fig. 5b. Via the above data, the linearity of the pressure sensor at room temperature was 0.16% FS, the hysteresis was 0.04% FS, the repeatability was 0.06% FS, the basic error was ±0.23% FS, accuracy was 0.18% FS. The full-scale output and sensitivity were 159.42 mV and 7.97 mV/MPa, respectively. Compared with the theoretical data, the errors were within 3%.

**Table 1:** Calibration date at 25 °C

Pressure (MPa)	Voltage output (mV)			
	1st Round		2nd Round	
	Upward	Downward	Upward	Downward
0	87.42	87.47	87.47	87.47
4	119.00	118.96	119.03	118.97
8	150.78	150.72	150.83	150.74
12	182.68	182.63	182.73	182.72
16	214.72	214.69	214.76	214.74
20	246.84	246.84	246.82	246.82

The zero output of the pressure unit was measured at room temperature. The sampling interval was 1 s, and the sampling time was 30 min. The output signal curve is shown in Fig. 5c. The zero-drift coefficient was calculated to be 0.04% FS utilising Eq. 5.

5 $${d}_{z}=({y}_{d\max }-{y}_{d\min })/{y}_{\rm{FS}}\times 100 \%$$

where *y*_*d*max_ is the maximum value in 30 min, and *y*_*d*min_ is the minimum value in 30 min.

The piezoresistive coefficient of silicon is a function of temperature, and the sensitivity of the pressure unit changes with temperature. Therefore, a different temperature environment was provided by a thermal chamber to test the sensitivity performance of the sensor at −40, 0, 40, 80, and 120 °C. The test was performed after holding for 30 min at each temperature to ensure temperature stability. The output values of the sensor at different temperatures are presented in Table 2, and Fig. 5d illustrates the corresponding characteristic curves. The sensitivities of the pressure unit in the integrated sensor at different temperatures were calculated, as illustrated in Fig. 5e.

**Table 2:** Calibration date at different temperatures

Pressure (MPa)	Voltage output (mV)				
	−40 °C	0 °C	40 °C	80 °C	120 °C
0	84.98	77.54	73.70	69.63	68.90
4	122.22	110.87	103.66	97.50	94.57
8	159.83	144.43	133.77	125.83	120.60
12	197.56	178.14	164.51	153.83	146.71
16	235.47	211.98	194.98	181.90	172.80
20	273.38	245.96	225.47	210.20	199.12

The temperature sensitivity drift of the pressure unit was calculated to be −0.19% FS by Eq. 6, and the sensitivity of the sensor gradually decreased with increasing temperature. Subsequently, the sensitivity of the pressure sensor was compensated according to this law.

6 $$\beta =\frac{{y}_{\rm{FS}}({t}_{2})-{y}_{\rm{FS}}({t}_{1})}{{y}_{\rm{FS}}({t}_{1})({t}_{2}-{t}_{1})}\times 100 \%$$

where *t*_1_ is the lowest temperature; *t*_2_ is the highest temperature; *y*_FS_(*t*_1_) is the full-scale output at the lowest temperature, and *y*_FS_(*t*_2_) is the full-scale output at the highest temperature.

The resistance of the temperature sensor was also tested in the temperature range of −40 to 120 °C, and the least square method was employed to fit it. The variations in platinum resistance at different temperatures are presented in Table 3.

**Table 3:** Resistance of temperature unit at different temperatures

T/°C	Test resistance/kΩ	Fitting resistance/kΩ	Resistance deviation/kΩ	Temperature deviation/°C
−40	1.395	1.394	−0.001	−0.275
0	1.595	1.594	−0.001	−0.196
40	1.791	1.794	0.003	0.683
80	1.993	1.995	0.002	0.363
120	2.197	2.195	−0.002	−0.355

The TCR obtained from Eq. 7^30^ was 3142.997 ppm/°C.

7 $${{TCR}}=\frac{{R}_{t2}-{R}_{t1}}{{R}_{t1}({t}_{2}-{t}_{1})}\times {10}^{6}$$

where *R*_*t*2_ is the resistance at *t*_2_; *R*_*t*1_ is the resistance at *t*_1_; *t*_1_ is 0 °C, and *t*_2_ is the highest temperature of the test. The data were fitted to a straight line, as illustrated in Fig. 5f, and the relationship between the platinum resistance and temperature is shown in Eq. 8.

8 $${R}_{T}=0.00501T+1.594$$

The calculated temperature sensor sensitivity was 5.01 Ω/°C, the nonlinearity was 0.16% FS, accuracy was 0.32% FS, and the measurement error was less than ±1 °C. The outputs of the temperature sensor under different pressures are shown in Fig. 5g. It can be seen that the pressure does not affect the output of the temperature sensor.

The indices of the temperature and pressure integrated sensors are listed in Table 4. Compared with the published literature, our integrated sensor chip had a small size, large pressure range, and high linearity and precision; however, there was still a gap compared with mature products. The temperature measurement unit also exhibited high sensitivity.

**Table 4:** Performance parameters comparison with published literature

Source	Chip size (mm)	Pressure range	Linearity	Basic error	Temperature range (°C)	TCR
Our work	1.8 × 1.8 × 0.75	0–20 MPa	0.16% FS	±0.23% FS	−40 to 120	3142.997 ppm/°C
Wang et al.^20^	2.5 × 2.5 × 0.84	0–450 kPa	0.84% FS	/	/	−578 ppm/°C
Xu et al.^21^	4.0 × 6.0 × 0.9	0–200 kPa	0.40% FS	/	−30 to 150	/
Mansoor et al.^31^	3.8 × 3.8	0–75 kPa	0.25% FS	/	20–300	/

## Conclusion

A leadless temperature and pressure integrated sensor based on TSV and Au–Au bonding processes is proposed. The pressure and temperature units are connected longitudinally via the TSV and Au–Au bonding processes, which reduces the lateral dimension and avoids signal interference between the two units. The pressure unit adopts a simple and reliable flat film structure, and applies pressure to the back, effectively reducing the pressure medium requirements. The addition of the fillet design increases the natural frequency. A platinum resistor with a stable chemical performance and high precision is selected as the temperature unit. Instead of conventional metal ball welding, a solder ball effectively improves the reliability of the electrical connection of the sensor. The chip is fabricated with a reasonable and reliable process and packaged as an integrated sensor. Finally, the sensitivity of the pressure unit in the integrated sensor is 7.97 mV/MPa in the range of 0–20 MPa, and the linearity is 0.16% FS. The hysteresis is 0.04% FS, the repeatability is 0.06% FS, the basic error is ±0.23%FS, the accuracy is 0.18% FS, the zero-time drift is 0.04% FS, and the thermal sensitivity drift is −0.20% FS at −40 to 120 °C. The TCR of the platinum resistor in the temperature unit is 3142.997 ppm/°C and the measurement error is less than ±1 °C.
